# Understanding Microbiome Stability in a Changing World

**DOI:** 10.1128/mSystems.00157-17

**Published:** 2018-03-20

**Authors:** Ashley Shade

**Affiliations:** a Michigan State University Department of Microbiology and Molecular Genetics and Department of Plant, Soil and Microbial Sciences, Michigan State Plant Resilience Institute, Program in Ecology, Evolutionary Biology, and Behavior, East Lansing, Michigan, USA

**Keywords:** Centralia, disturbance ecology, diversity reservoir, dormancy, environmental change, microbial ecology, rare biosphere, stability, structure-function, temporal dynamics

## Abstract

Microbiomes underpin biogeochemical processes, sustain the bases of food webs, and recycle carbon and nutrients. Thus, microbes are frontline players in determining ecosystem responses to environmental change. My research team and I investigate the causes and consequences of microbiome stability. Our primary objective is to understand the responses of complex microbiomes to stressors associated with environmental change. This work is important because Earth is changing rapidly and drastically, and these changes are expected to have serious consequences for ecosystems, their inhabiting organisms, and their microbiomes. Therefore, we aim to understand the repercussions of alterations to microbiome structure and functions and to use this information to predict the responses of microbiomes to stressors. This research is critical to prepare for, respond to, and potentially moderate environmental change. We anticipate that the results of our research will contribute toward these goals and will broadly inform management or manipulation of microbiomes toward desired functions.

## Abstract (precis)

Microbiomes underpin biogeochemical processes, sustain the bases of food webs, and recycle carbon and nutrients. Thus, microbes are frontline players in determining ecosystem responses to environmental change.

## PERSPECTIVE

It is not a trivial task to study microbiome stability, as a microbiome is a complex system within itself that also engages in intricate feedbacks with its ecosystem. Furthermore, environmental change is complex and encompasses interrelated and sometimes compounded stressors, including increased temperatures and greenhouse gases, land-use changes, and higher frequencies and intensities of extreme weather events. A multifaceted tactic for understanding microbiome stability is required, inclusive of both systems-level and mechanistic approaches. It is also important to consider the temporal dynamics of the microbiome and ecosystem. Given this complexity, my lab advances multiple research directions. Our toolbox includes high-throughput omics (mainly sequencing and metabolomics), *in situ* field surveys and experiments, synthetic microbial communities (1), and quantitative methods in environmental microbiology and microbial isolation and characterization (e.g., reference 2). In addition, we ground our microbiome work in ecological theory and seek to identify and understand the ecological principles that explain overarching patterns across communities and ecosystems (e.g., references 3 and 4). Thus, though we currently are focused on projects investigating microbiomes associated with plants and soils, we intend that our research will contribute to predictive, cross-ecosystem advances in microbiome ecology. In this piece, I highlight only one of our research directions about which I am particularly excited.

### A model ecosystem for interrogating the dynamics of diversity reservoirs and their contributions to microbiome stability.

Many environmental and host-associated microbiomes are species rich and harbor immense phylogenetic and functional biodiversity. However, within microbiomes, most biodiversity exists as a reservoir that is comprised of both rare and dormant microbial populations. Though some rare taxa are transient, many rare taxa are persistent over time and contribute to a microbiome’s standing biodiversity. Similarly, dormant microbial taxa offer a reservoir of diversity and functions that persist despite conditions unfavorable for their growth. Notably, there is expected to be some yet-unquantified redundancy between these two categories, as limited by observation methods (i.e., some members detected in low abundance may also be dormant).

Diversity reservoirs have ecoevolutionary consequences for microbiomes, as they may persist given stressful conditions and contribute to the microbiome and its genetic pool after an unknown but potentially indefinite passage of time. More importantly, there is evidence that taxa from diversity reservoirs can offer functions and mediate processes that have consequences for ecosystem performance. However, we do not understand the contributions of rare taxa and dormant taxa for community stability. System-specific studies collectively suggest a robust response of rare and dormant taxa to stressors. Thus, we hypothesize that the dynamics and interactions of microbial diversity reservoirs are key for understanding community stability outcomes.

**FIG 1:**
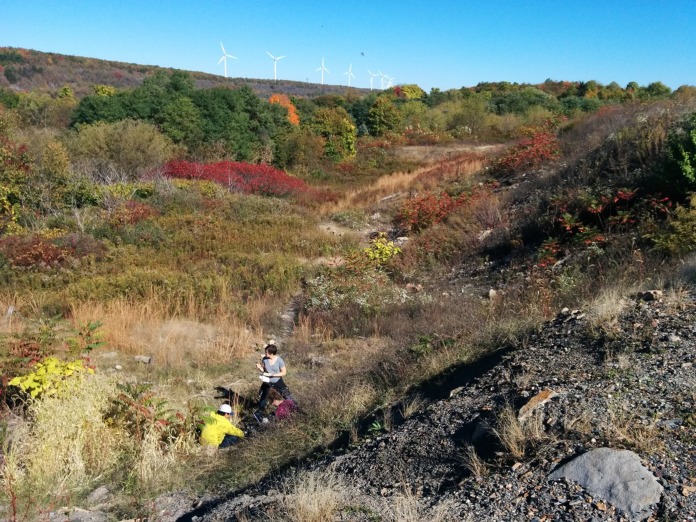
The Shade research team sampling soil above the coal seam fire, Centralia, PA, in October 2015.

Because many stressors are transient and the temporal scale of microbial responses is difficult to discern, identifying a good *in situ* system to test hypotheses about the contributions of rare and dormant taxa to stability is challenging. An ideal system may include a press disturbance that has a clear endpoint but also provides an extended opportunity to interrogate rare-to-prevalent dynamics and transitions into and out of dormancy. Thus, we have adopted a model *in situ* system that fortuitously meets these criteria: the terrestrial ecosystem overlying the Centralia, PA, coal seam fire (Fig. 1). The Centralia fire has been burning since 1962 and is estimated to continue to burn until its fuel is exhausted, potentially for another 100 years. The fire currently underlies more than 150 acres and continues to spread 3 to 7 m/year through underground coal seams (5). As the fire expands into new areas, it also retreats from some affected sites, which then recover to ambient temperatures (5–7). Thus, the end of the stressor is delineated by temperature, and a chronosequence of fire-affected soils provides a gradient of disturbance response and recovery over space and time. This unusual habitat provides a unique opportunity to investigate the impact of a press stressor on soil microbiome stability and ecosystem processes (e.g., reference 8) and allows us to probe the biological limits of resistance and resilience after an extreme stressor.

Centralia is particularly suited for investigations of rare and dormant taxa. We can interrogate there the population dynamics and activities of typically rare and dormant taxa that are responsive to fire (9) and compare fire-affected to recovered and yet-unaffected soil communities. There is a clear benefit of the Centralia fire chronosequence for comparative analysis; otherwise, the dynamics of typically rare and dormant taxa may be masked by those of prevalent populations or occur at temporal scales that could be missed. The slow progression and extreme conditions of the fire together offer an opportunity to quantify ecoevolutionary dynamics of diversity reservoirs and to understand their contributions to microbiome stability and ecosystem processes.

Capitalizing on the Centralia chronosequence, we were surprised to find that soil microbiomes reasonably recover in structure after the fire subsides. Fire-affected soils had divergent community structures despite shared measured physicochemical conditions, and we hypothesized that local differences in resuscitation of dormant members may in part explain the divergence (10). Our immediate future directions are to determine the composition and structure of the dormant soil microbiome, to understand their resuscitation dynamics given fire, and to quantify the contributions of rare and dormant members to functions. So far, we have evidence that members of the rare biosphere contribute to arsenic resistance (2) and therefore can have consequences for arsenic transformation in Centralia. We also found that the diverse taxa inhabiting fire-affected soils, on average, have smaller genomes, fewer two-component regulatory systems, and fewer antibiotic resistance and production genes (11; J. W. Sorensen, T. K. Dunivin, T. C. Tobin, and A. Shade, submitted for publication). These findings are our first steps in parsing the ecological roles of previously rare and dormant members for the Centralia soil microbiome, and this work is ongoing in my lab. We also are interested in the relative contributions of contemporarily versus historically “banked” dormant cells to the dormant reservoir and the implications of this for stability. We expect that routine observation of the chronosequence as the fire advances will inform microbiome response and recovery at each sample site, allowing us to probe metacommunity aspects of stability and to link them to the dynamics of rare and dormant taxa.

In conclusion, we expect that studying the soil microbiomes in Centralia, a model ecosystem impacted by long-term disturbance, will be fruitful for understanding the roles of diversity reservoirs for microbiome resilience and for understanding the general ecology and mechanisms of microbiome stability.

### Projections for the next 5 years.

In the next 5 years, I project that environmental microbiome analysis will begin to be informed by the complete range of metabolic activity of its members. Instead of just extracting and then extrapolating from bulk DNA or RNA, microbial ecologists will better distinguish between active and inactive members within environmental microbiomes and even attempt to quantify the entire spectrum of cellular metabolic activity. There also will be efforts to quantify contributions of metabolically active-but-not-dividing cells to community functions. Because exponential growth in many environments is atypical, microbial ecologists will begin to account for differences in growth and activities of taxa to predict consequences of biotic interactions like competition, antagonism, and synergy.

I also project rapid progress in the next 5 years in efforts to identify and manipulate core microbiomes. From complex environments, core microbiomes comprised of 10 to 100 members will be assembled in the laboratory and manipulated to determine their emergent properties and hone their important functions. These synthetic microbial communities will be developed and applied for use in agriculture, conservation, human and animal health, and bioengineering.

I expect advances in the use of (meta-)metabolomics for understanding microbiome functions, as the field advances to consider the ecology of microbial ecochemistry for biotic interactions *in situ*. The technical leaps in sensitivity and throughput of mass spectrometry methods of today are analogous to those of sequencing in the early 2000s, and it will become more accessible for individual labs to generate and analyze these types of data. There will be improvements in understanding secondary metabolism and the roles of extracellular molecules and signals in determining microbial functions and biotic interactions. This will inform structure-function relationships in microbiome ecology and promote understanding of the temporal and spatial scales over which microorganisms can interact.

Finally, it is my hope that, as a field, we will continue to promote and curate open-source workflows and data. Sharing of high-quality digital data and analyses promotes reproducibility, strengthens science, and democratizes research.
